# Superstable homogeneous lipiodol–ICG formulation: initial feasibility and first-in-human clinical application for ruptured hepatocellular carcinoma

**DOI:** 10.1093/rb/rbac106

**Published:** 2022-12-16

**Authors:** Yongfu Xiong, Pan He, Yang Zhang, Hu Chen, Yisheng Peng, Peng He, Jie Tian, Hongwei Cheng, Gang Liu, Jingdong Li

**Affiliations:** Department of Hepatobiliary Surgery, Academician (Expert) Workstation, Affiliated Hospital of North Sichuan Medical College, Nanchong 637600, China; State Key Laboratory of Molecular Vaccinology and Molecular Diagnostics, Center for Molecular Imaging and Translational Medicine, School of Public Health, Xiamen University, Xiamen 361102, China; Department of Hepatobiliary Surgery, Academician (Expert) Workstation, Affiliated Hospital of North Sichuan Medical College, Nanchong 637600, China; State Key Laboratory of Molecular Vaccinology and Molecular Diagnostics, Center for Molecular Imaging and Translational Medicine, School of Public Health, Xiamen University, Xiamen 361102, China; Nuclear Medicine and Molecular Imaging Key Laboratory of Sichuan Province, Luzhou 646000, China; State Key Laboratory of Molecular Vaccinology and Molecular Diagnostics, Center for Molecular Imaging and Translational Medicine, School of Public Health, Xiamen University, Xiamen 361102, China; State Key Laboratory of Molecular Vaccinology and Molecular Diagnostics, Center for Molecular Imaging and Translational Medicine, School of Public Health, Xiamen University, Xiamen 361102, China; State Key Laboratory of Molecular Vaccinology and Molecular Diagnostics, Center for Molecular Imaging and Translational Medicine, School of Public Health, Xiamen University, Xiamen 361102, China; State Key Laboratory of Molecular Vaccinology and Molecular Diagnostics, Center for Molecular Imaging and Translational Medicine, School of Public Health, Xiamen University, Xiamen 361102, China; Key Laboratory of Molecular Imaging, Institute of Automation, Chinese Academy of Sciences, Beijing 100190, China; State Key Laboratory of Molecular Vaccinology and Molecular Diagnostics, Center for Molecular Imaging and Translational Medicine, School of Public Health, Xiamen University, Xiamen 361102, China; State Key Laboratory of Molecular Vaccinology and Molecular Diagnostics, Center for Molecular Imaging and Translational Medicine, School of Public Health, Xiamen University, Xiamen 361102, China; Department of Hepatobiliary Surgery, Academician (Expert) Workstation, Affiliated Hospital of North Sichuan Medical College, Nanchong 637600, China

**Keywords:** conversion therapy, fluorescent navigation, hepatectomy, spontaneous tumor rupture

## Abstract

The most common treatment of spontaneous tumor rupture hemorrhage (STRH) is transcatheter arterial embolization (TAE) followed by liver resection, and surgical navigation using near-infrared fluorescence is effective method for detecting hidden lesions and ill-defined tumor boundaries. However, due to the blockage of the tumor-supplying artery after effective TAE treatment, it is difficult to deliver sufficient fluorescent probes to the tumor region. In this study, we report on the successful application of superstable homogeneous intermixed formulation technology (SHIFT) in precise conversion hepatectomy for ruptured hepatocellular carcinoma (HCC). A homogeneous lipiodol–ICG formulation obtained by SHIFT (SHIFT-ICG) was developed for clinical practice for STRH. A ruptured HCC patient received the combined protocol for embolization and fluorescence surgical navigation and exhibited excellent hemostatic effect. Lipiodol and ICG were both effectively deposited in the primary lesion, including a small metastatic lesion. In follow-up laparoscopic hepatectomy, SHIFT-ICG could clearly and precisely image the full tumor regions and boundaries in real time, and even indistinguishable satellite lesions still expressed a remarkable fluorescence intensity. In conclusion, the simple and green SHIFT-ICG formulation can be effectively used in emergency embolization hemostasis and later precise fluorescence navigation hepatectomy in patients with ruptured HCC bleeding and has high clinical application value.

## Graphical Abstract

**Figure:**
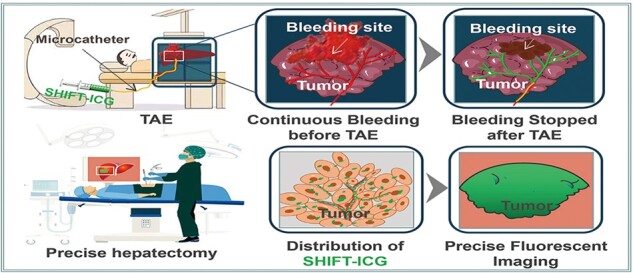

## Introduction

Hepatocellular carcinoma (HCC) is the most common primary liver cancer and is a significant cause of cancer-related mortality [1]. The presentations vary substantially, ranging from asymptomatic early-stage focal malignancies to diffuse infiltrative disease [2]. A less common presentation is spontaneous tumor rupture hemorrhage (STRH). Recently, the reported incidence rate of the spontaneous rupture of HCC has varied from 2.3% to 5.9% [3–5]. Although relatively infrequent, the high acute mortality rate associated with ruptured HCC makes spontaneous rupture the third leading cause of HCC-associated mortality worldwide. With the development of surgical techniques such as hepatic resection, angiographic intervention and critical care medicine, various treatment modalities for ruptured HCC have been introduced, including conservative medical management, surgery and transcatheter embolization [6]. Among these, transcatheter arterial embolization (TAE) followed by liver resection is the most commonly used treatment for STRH [7]. However, the unclear boundaries between tumor and normal liver tissue and hidden minimal lesions have severe limited liver resection after long-term TAE hemostatic treatment for STRH.

A promising method for this situation is fluorescent surgical navigation. Indocyanine green (ICG) is the most commonly used clinical fluorescent imaging agent. In recent studies, ICG has become increasingly important in identifying HCC during hepatectomy [8, 9]. However, in traditional fluorescence-guided hepatectomies, ICG has been used intravenously 3–5 days before surgery. ICG is a small molecule with limited stability, the fluorescence performance is reduced due to agglomeration quenching [10]. Particularly, owing to blockage of the tumor-feeding artery following TAE, it is difficult to deliver ICG to the tumor region. As a result, surgical resection after TAE conversion therapy cannot be implemented effectively and accurately.

**Figure 1.**
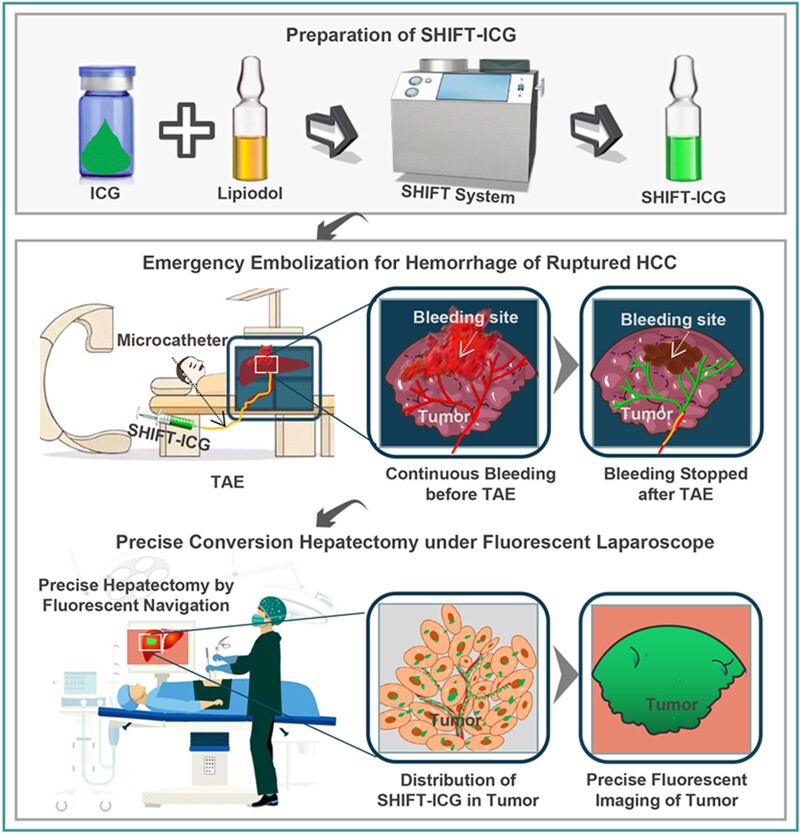
An overview of the preparation and research processes of SHIFT-ICG. The system of SHIFT was used to make SHIFT-ICG formulation under supercritical carbon dioxide (SC-CO_2_). Subsequently, SHIFT-ICG deposited in the tumor-feeding artery through TAE, and then achieving acute hemostatic therapy and subsequent precise fluorescence-guided resection.

To deal with this issue, we presented a technique called ‘superstable homogeneous intermixed formulation technology’ (SHIFT) for the homogeneous physical mixing of a lipiodol and ICG formulation (called SHIFT-ICG). The SHIFT-ICG is very suitable for fluorescence-navigated liver resection following long-term TAE conversion [11–14]. This study reports a successfully treated case of ruptured HCC by precise conversion hepatectomy after TAE emergency treatment with SHIFT (Fig. 1).

## Materials and methods

### Materials

Jiangsu Hengrui Pharmaceutical Co., Ltd (Jiangsu, China) provided the lipiodol. Dandong Medical and Pharmaceutical Co., Ltd (Dandong, China) supplied indocyanine green for the study. Oil red O was purchased from Wuhan Servicebio Biotechnology Co., Ltd (Wuhan, China).

### Construction of the superstable SHIFT-ICG pharmaceutics

First, 10 mg ICG and 10 ml lipiodol were added to the clean reactor of SHIFT developed by our group. Second, the reaction pressure was set at 20 MPa, temperature at 35°C and stirring speed at 1000 rpm. In this state, carbon dioxide (CO_2_) reached a supercritical state [15]. After 1 h incubation, the product was compiled with the 5000 rpm centrifugation to remove impurities and sterilized by irradiation. Rotational rheometer was applied to test the viscosity with increasing shear rate. And the imaging capability of formulation was acquired by digital subtraction angiography (DSA), and the gray intensity was quantified. The ICG release was achieved by co-incubation with saline (0.9%), and determination of ICG in saline was subjected to measure the ICG release rate *in vitro*.

### Transarterial embolization

Under local anesthesia, the femoral artery was punctured, and the celiac trunk and superior mesenteric arteries were angiographed. After determining the feeding artery and the site of bleeding, the microcatheter was used to enter the target artery superselectively. Afterwards, SHIFT-ICG formulation was injected at an ICG concentration of 1 mg/ml after identifying the bleeding site. The injected dose of SHIFT-ICG formulation depends on the tumor size until the target artery blood stasis or regurgitation was achieved.

### Fluorescent laparoscopic hepatectomy

The fluorescent laparoscopic system (FLS) was used for observing the suspicious sites in advance of tumor resection with the camera's head 10–20 cm away. After resection of the lesion, the FLS was used to examine whether fluorescence remained in the resected site or surgical specimens. After resection, all specimens were pathologically examined.

### Ethical approval

Research procedures involving humans were conducted under the ethical guidelines of the institution and the national research committee and the 1964 Declaration of Helsinki and its later amendments or comparable ethical standards. North Sichuan Medical College's Institutional Review Board approved this study and registered it with the Chinese Clinical Trial Registry, and the registration number is ChiCTR2000035055.

## Results

### *Construction of a superstable homogeneous lipiodol*–*ICG formulation*

**Figure 2.**
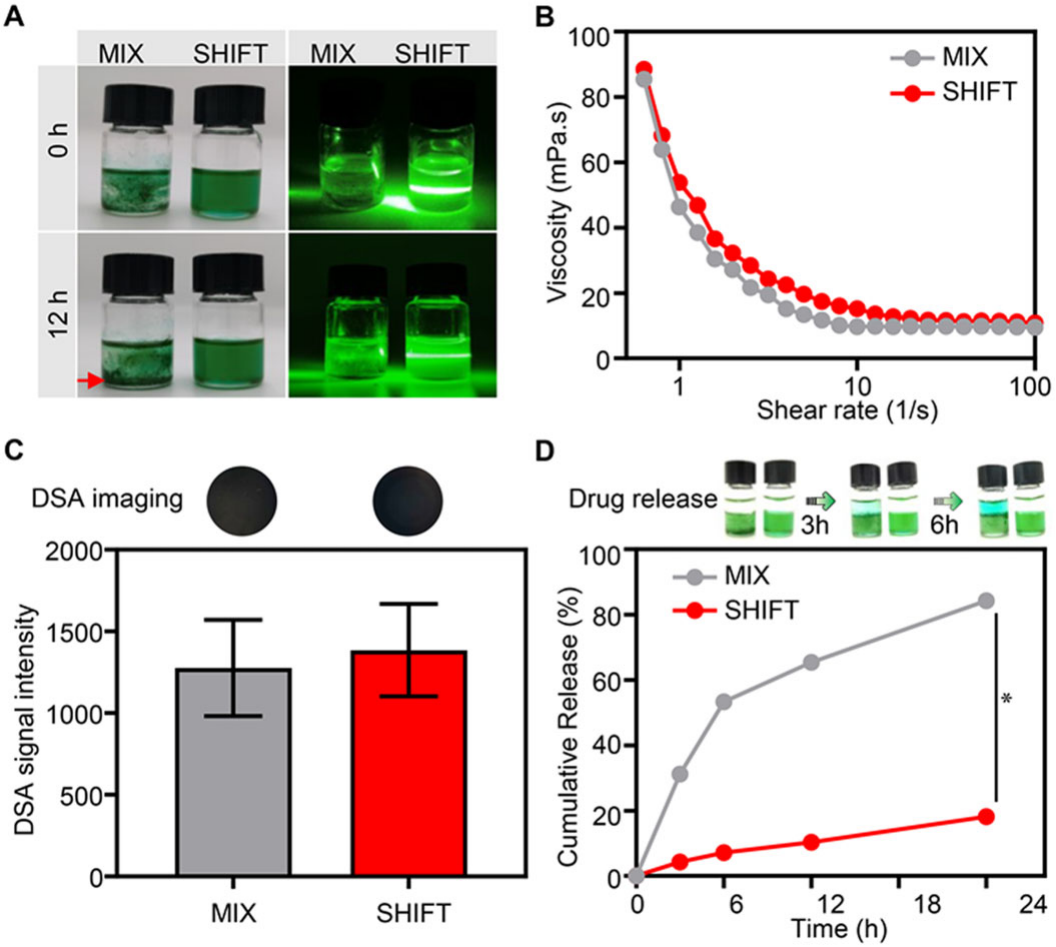
Construction of a superstable homogeneous lipiodol–ICG formulation. (**A**) The photograph of the two formulations with conventional three-way stopcock (MIX) and SHIFT methods. (**B**) The viscosity value was measured by rheology test. (**C**) DSA imaging and the relative signal intensity, the two-tailed *t*-test was subjected to compare the statistical difference between two groups and *P* values were 0.67, which was considered no significant statistical difference. (**D**) The cumulative ICG release of the two formulations (MIX and SHIFT groups) at indicated times, two-way analysis of variance was subjected to test the statistical difference between two groups at different time. *P <* *0.05 was considered significant statistical difference.

Conventional drug dispersion in interventional therapy is achieved by a three-way stopcock, and the unstable drug dispersion has always been a key factor that restricts the clinical prognosis. Considering the good solubility of supercritical carbon dioxide, we reported a superstable homogeneous iodinated formulation technology. In this study, a conventional three-way stopcock was used as a control (the MIX group). As shown in Fig. 2A, ICG was fully dispersed in the lipiodol with SHIFT, and the MIX group displayed poor ICG dispersion. The light scattering capability of the SHIFT group also showed the Tyndall effect. In contrast, significant ICG subsidence appeared in the MIX group after 12 h, and there was no Tyndall effect due to its inhomogeneous features. The viscosity of the embolic agent is a key factor for the therapeutic performance and injectable capability, so a rheological analysis was performed on the two formulations. The results demonstrated that the formulations’ viscosities decreased with the increase of the shear rate, indicating good shear-shinning properties (Fig. 2B). DSA imaging capability of the lipiodol was also confirmed, as shown in Fig. 2C. The quantitative analysis revealed that the SHIFT treatment did not decrease the imaging performance of lipiodol. Furthermore, the results of ICG drug release also confirmed that the SHIFT-ICG formulation with SHIFT treatment demonstrated a sustained drug-release capability. However, the conventional MIX group showed rapid drug release due to unstable drug dispersion in the lipiodol (Fig. 2D). The sustained drug-release behavior of SHIFT-ICG formulation was also confirmed in our previous *in vivo* studies about fluorescence surgery after long-term TAE conversion therapy.

### Admission status and treatment of cases

**Figure 3.**
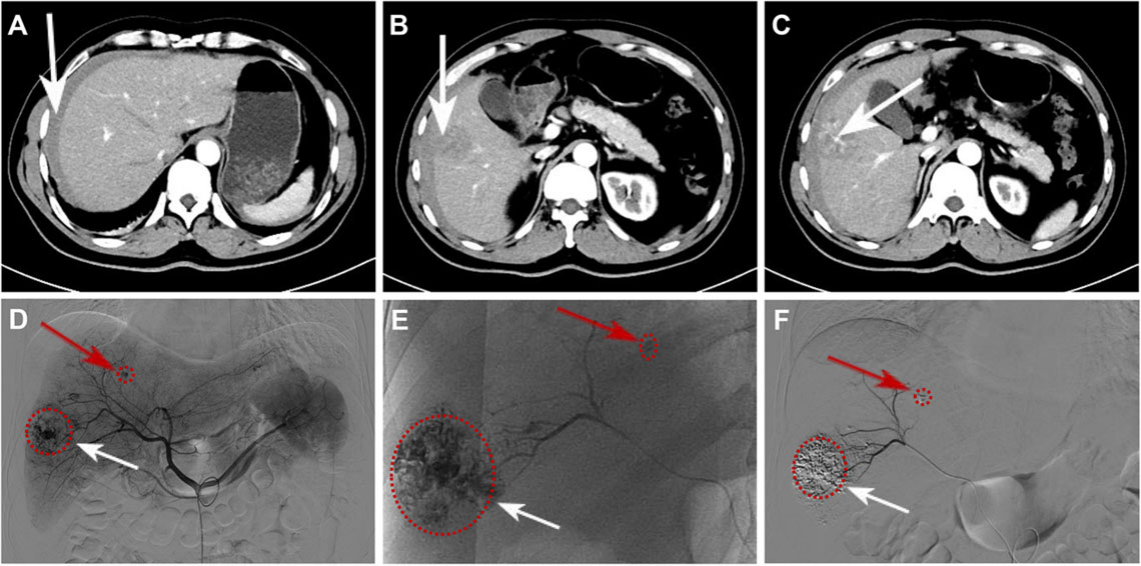
The computed tomography and TAE findings. (**A–C**) The right lobe of the liver was observed to have a tumor measuring 4.1 × 3.5 cm^2^ in diameter, and bloody ascites was also observed. The situation with the DSA angiography and SHIFT-ICG embolization: (**D**) before embolization, (**E**) during embolization and (**F**) after embolization. White arrow: primary lesions, red arrow: small metastatic lesion.

A 51-year-old male patient with hepatitis B infection and cirrhosis underwent abdominal ultrasonography due to swelling and pain in the upper right abdomen. He was diagnosed with changes in chronic liver disease and abdominal pelvic effusion. An upper abdominal enhanced computed tomography (CT) scan showed abdominal cavity effusion (Fig. 3A) and a solitary liver tumor measuring 4.1 × 3.5 cm^2^ located in segment VIII (Fig. 3B) with ruptured hemorrhage (Fig. 3C). Subsequently, TAE with SHIFT-ICG prepared by SHIFT combined with hepatectomy was used for treatment. During the implementation of TAE, it was found that DSA imaging could clearly locate the primary bleeding lesion (Fig. 3D, white arrow). Furthermore, a new micro-metastatic lesion was found, which had not been detected using CT (Fig. 3D, red arrow). The SHIFT-ICG formulation effectively deposited in the primary lesion (Fig. 3E and F, white arrow) and even in the small metastatic lesion (Fig. 3E and F, red arrow), with excellent performance of embolization and hemostasis. The white blood cell, alanine transaminase and aspartate transaminase indexes of patients with TAE before and 3 days after operation were 23.1 vs 12.70 10E9/l, 96 vs 87 U/l and 44 vs 78 U/l, respectively. The results showed that SHIFT-ICG formulation not only did not increase the systemic response of patients but also improved the inflammatory reaction and liver function damage caused by acute bleeding, suggesting that SHIFT-ICG formulation has high biological safety.

### Effect of fluorescein-guided hepatectomy after conversion therapy

**Figure 4.**
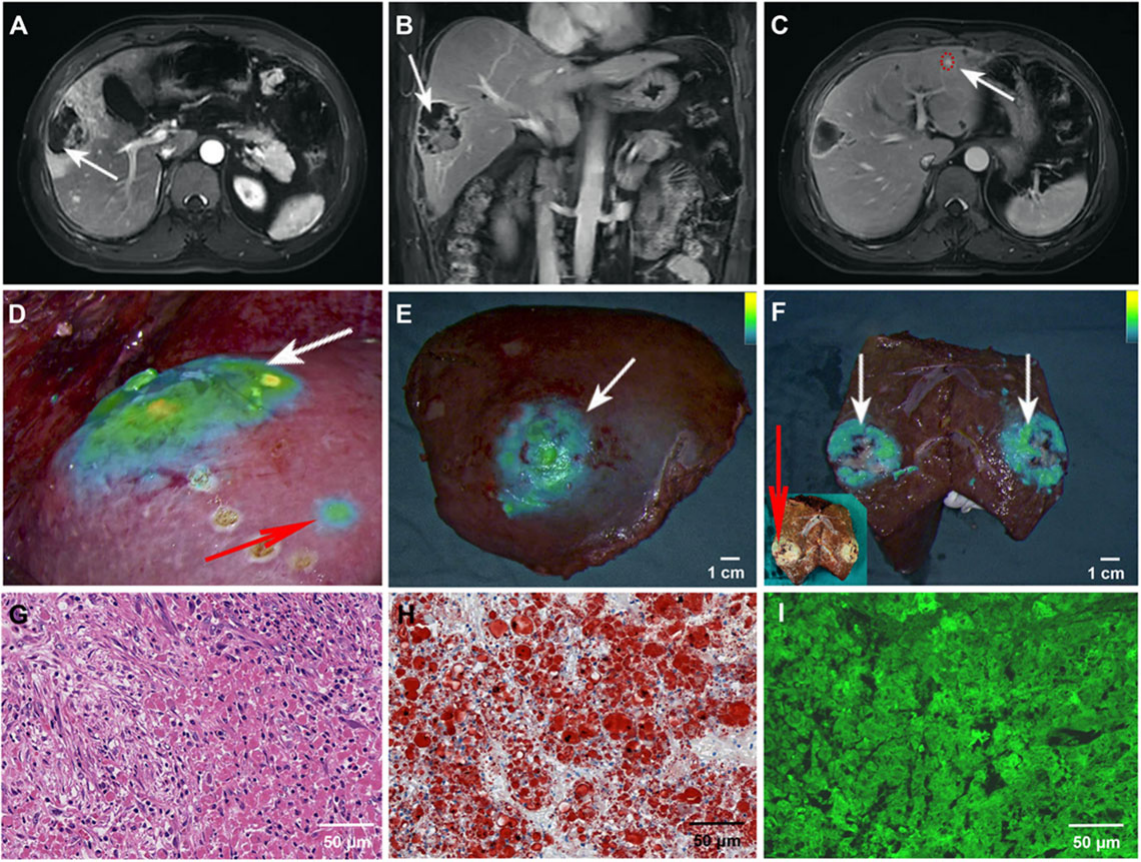
The surgical navigation effect of SHIFT-ICG after successful conversion. The tumor state before surgical resection was measured by magnetic resonance imaging: (**A**) coronal plane, (**B**) horizontal plane and (**C**) small metastatic lesion. (**D**) Fluorescence imaging before surgical resection (white arrow: primary tumor lesions; red arrow: small metastatic lesion). (**E**) Whole resected tumor lesions imaged by fluorescence. (**F**) An image showing the resected liver tumor lesions (red arrow) and the fluorescence imaging (white arrow). Postoperative histological examination: (**G**) H&E staining was subjected to evaluate the pathological evaluation, (**H**) Oil red staining was used to test the lipiodol deposition and (**I**) ICG retention in tumor cells was observed by fluorescence confocal microscopy imaging.

One week after TAE, magnetic resonance imaging showed that the primary HCC lesion was necrotic, and the perihepatic hematoma had disappeared (Fig. 4A and B, white arrow). The small metastases were clearly visible without enlargement (Fig. 4C) and reached the standard for radical surgical resection. We subsequently performed fluorescent laparoscopic hepatectomy. Intraoperatively, it was found that SHIFT-ICG could accurately locate the location and boundary of primary HCC lesions (Fig. 4D, white arrow) and small metastases (Fig. 4D, red arrow) with excellent fluorescence performance after long-term TAE therapy, which was also verified in *in vitro* tumor specimens (Fig. 4E). Observation of the dissected tumor specimens revealed that SHIFT-ICG was largely deposited in tumor lesions (Fig. 4F, red arrow), so the fluorescence signal could accurately reveal the whole lesion (Fig. 4F, white arrow). The tumor specimens were confirmed by hematoxylin–eosin (H&E) staining as HCC (Fig. 4G). Oil red O staining showed a large amount of lipiodol deposition in the tumor tissue (Fig. 4H). The whole tumor tissue contained a stronger ICG fluorescence signal (Fig. 4I).

### Postoperative treatment and follow-up

**Figure 5.**
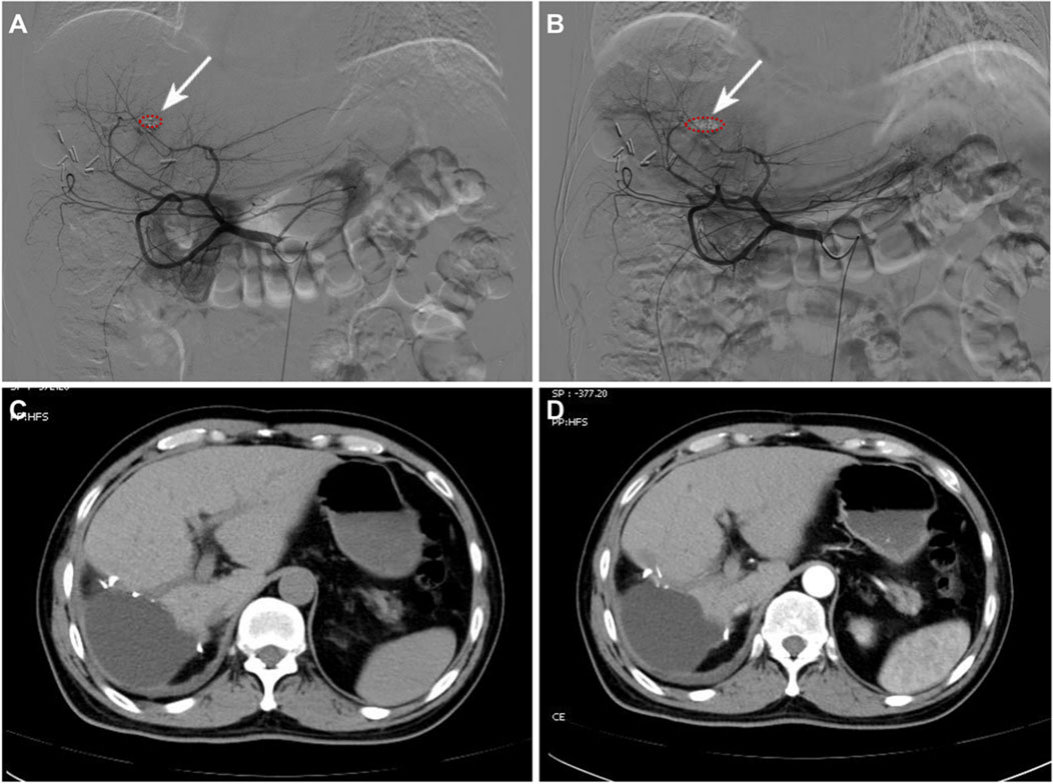
Follow-up treatment and re-examination. (**A**) One week after hepatectomy, the patient received the angiographic imaging. (**B**) The angiographic images of the patient with the treatment of infusion chemotherapy and TACE. The contrast-enhanced CT of the patient after hepatectomy three months: (**C**) venous phase and (**D**) arterial phase.

For small metastases, we performed transhepatic arterial infusion chemotherapy and transhepatic arterial chemotherapy and embolization (TACE) again at 1 and 4 weeks after the surgery to prevent liver failure caused by residual liver insufficiency (Fig. 5A and B). Three months after the surgery, a regular contrast-enhanced CT examination showed that the patient's right liver had no residual tumor tissue after the surgery, and the metastases of the liver had disappeared (Fig. 5C and D). At the same time, the alpha-fetoprotein level returned to normal (<200 µg/l) from 5012.60 µg/l. These results suggested that TAE with SHIFT-ICG combined with fluorescent surgical resection had an excellent effect and good prospects for clinical application for patients with liver cancer rupture and hemorrhage.

## Discussion

The long-term oncological outcome should be considered in managing ruptured HCC in addition to acute-phase management dealing with bleeding issues. The management approach comprises two steps, TAE and surgical resection, to achieve hemostasis and stabilization of the patient first and then to complete the treatment with oncological surgery [7, 16]. However, the recurrence rate is still high after TAE conversion therapy and even after successful hepatectomy [17]. The higher recurrence rate might contribute to the spread of cancer cells due to tumor rupture, as well as tumor boundaries and microscopic metastases that are difficult to recognize.

As a safe reagent, ICG is used in clinical practice to assess hepatobiliary function, liver and gastric blood flow and cardiac output, among other things. Fluorescence imaging of ICG for the detection of HCC or metastases has been widely demonstrated [18, 19]. However, its application in hepatectomy after embolization transformation therapy is rarely reported. The main reason is that the conventional intravenous injection of ICG after interventional embolization conversion therapy cannot solve the problem of the effective distribution of ICG [20, 21].

Therefore, we proposed a fluorescence surgery navigation strategy after long-term TAE conversion therapy using fluorescent probe and lipiodol formulation. However, to achieve this goal, the first problem to solve was that hydrophilic ICG drugs cannot disperse in oily substances [22, 23]. In this study, by using simple, green and economical SHIFT system, we were able to overcome the obstacles posed by traditional methods related to ICG molecules. By developing SHIFT, we were able to stabilize ICG in lipiodol and improve the condition of fluorescence surgery after long-term TAE conversion therapy [11, 14]. This was the first clinical report on the use of SHIFT-ICG formulation for emergency treatment and precise conversion hepatectomy of ruptured HCC. In our STRH cases, SHIFT-ICG had excellent embolization and hemostatic effects, and ICG molecules maintained long-term stability, so the whole tumor lesion and even micro-metastasis were revealed in the laparoscopic surgical resection 1 week later. This rapid and accurate identification of tumors ensured the complete resection of tumor tissue, greatly reduced the time of tumor detection in routine surgery, avoided excessive intraoperative bleeding and large-area trauma and improved the prognosis of patients. In addition, SHIFT-ICG can be used for accurate preoperative TAE-assisted fluorescence navigation for surgical resection of large or small HCC, and chemotherapeutics and radiopharmaceuticals [20, 24] can be enhanced by SHIFT.

## Conclusion

The simple and green SHIFT-ICG formulation, with excellent embolism and fluorescence imaging performance, can be effectively used in emergency embolization hemostasis and later precise fluorescence navigation hepatectomy in patients with ruptured HCC bleeding and has high clinical application value.
